# A combined long-range phasing and long haplotype imputation method to impute phase for SNP genotypes

**DOI:** 10.1186/1297-9686-43-12

**Published:** 2011-03-10

**Authors:** John M Hickey, Brian P Kinghorn, Bruce Tier, James F Wilson, Neil Dunstan, Julius HJ van der Werf

**Affiliations:** 1 School of Environmental and Rural Science, University of New England, Armidale, Australia; 2 Animal Genetics and Breeding Unit, University of New England, Armidale, Australia; 3 Centre for Population Health Sciences, University of Edinburgh, Teviot Place, Edinburgh, EH8 9AG Scotland; 4 School of Science and Technology, University of New England, Armidale, Australia; 5 Cooperative Research Centre for Sheep Industry Innovation, Armidale, Australia

## Abstract

**Background:**

Knowing the phase of marker genotype data can be useful in genome-wide association studies, because it makes it possible to use analysis frameworks that account for identity by descent or parent of origin of alleles and it can lead to a large increase in data quantities via genotype or sequence imputation. Long-range phasing and haplotype library imputation constitute a fast and accurate method to impute phase for SNP data.

**Methods:**

A long-range phasing and haplotype library imputation algorithm was developed. It combines information from surrogate parents and long haplotypes to resolve phase in a manner that is not dependent on the family structure of a dataset or on the presence of pedigree information.

**Results:**

The algorithm performed well in both simulated and real livestock and human datasets in terms of both phasing accuracy and computation efficiency. The percentage of alleles that could be phased in both simulated and real datasets of varying size generally exceeded 98% while the percentage of alleles incorrectly phased in simulated data was generally less than 0.5%. The accuracy of phasing was affected by dataset size, with lower accuracy for dataset sizes less than 1000, but was not affected by effective population size, family data structure, presence or absence of pedigree information, and SNP density. The method was computationally fast. In comparison to a commonly used statistical method (fastPHASE), the current method made about 8% less phasing mistakes and ran about 26 times faster for a small dataset. For larger datasets, the differences in computational time are expected to be even greater. A computer program implementing these methods has been made available.

**Conclusions:**

The algorithm and software developed in this study make feasible the routine phasing of high-density SNP chips in large datasets.

## Background

Knowing the phase of marker genotype data can be useful in genome-wide association studies (GWAS), because it makes it possible to use analysis frameworks that account for identity by descent (IBD) or parent of origin of alleles [1] and it can lead to a large increase in data quantities via genotype or sequence imputation e.g. [2,3]. Phasing entire genomes in GWAS datasets has been a computational bottleneck due to the unavailability of robust heuristic phasing methods when a family structure does not exist and to the computationally intensive nature of statistically based phasing methods, e.g. fastPHASE [4] and Beagle [5].

Long-range phasing (LRP) is a fast and accurate heuristic method for phasing of marker genotypes, which uses information from both related and seemingly unrelated individuals by invoking the concepts of surrogate parents and Erdös numbers, as defined in Kong et al. [6]. In comparison to phasing methods based on statistical inference, LRP has been reported to be 1,000 times faster with a 34% lower error rate than fastPHASE [6]. However, while LRP is powerful and efficient, the method is not fully robust. Applying LRP can result in parts of a given dataset being not phased or phased incorrectly. Incorrect identification of surrogate parents leads to incorrect phasing and this can occur when there is insufficient combinatorial power in the data to correctly identify, partition, or eliminate surrogate parents, or when genotyping errors exist. For any given locus, failure to find information pertaining to phase within the surrogate parents of an individual results in the locus being unphased.

The objective of this research was to expand the LRP algorithm of Kong et al. [6] in several ways to improve its robustness and ability to access more of the information contained within a dataset. We use the term long-range phasing and haplotype library imputation (LRPHLI) for the expanded algorithm, combining the concept of long-range phasing (LRP) and haplotype library imputation (HLI). In order to handle insufficient combinatorial power and errors in genotypes or physical maps, LRPHLI uses information from multiple surrogate parents and a more robust definition of surrogacy using the concepts of cores and tails (defined below). The LRPHLI partitions surrogate parents into paternal/maternal surrogates with or without pedigree information. A method to impute phase by HLI was developed to increase the overall phasing yield, particularly when the information pertaining to phase within the surrogate parents of an individual is insufficient. The haplotype library corrects mistakes created by false surrogate definition and/or genotyping errors. The performance of the method was evaluated using simulated data on both simulated and real pedigree structures as commonly found in livestock and human populations, with varying family sizes, depths of pedigree, historical effective population sizes, and SNP density. It was also evaluated using real human and livestock datasets. The method has been implemented in a new software package called AlphaPhase.

The sections that follow describe the LRP method of Kong et al. [6], the improvements made to LRP, the method of HLI, the simulated and real datasets that were used to test different aspects of the LRPHLI algorithm, and finally the performance of the LRPHLI algorithm.

## Methods

### Long-range phasing

**Figure 1:**
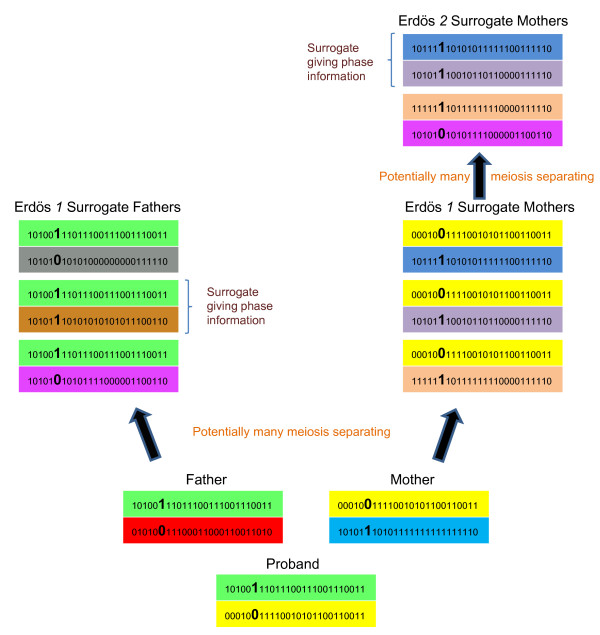
**Illustration of the long range phasing process**.

**Figure 2:**
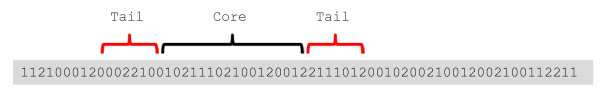
**A core and its adjacent tails**.

The LRP method of Kong et al. [6] is illustrated in Figure 1. These authors have suggested to phase a string of consecutive SNP in a single genome region (termed a core in our algorithm) by first identifying surrogate parents of each proband. Surrogate parents are individuals who share a haplotype with the proband and are identified as those individuals that do not have any opposing homozygote genotypes with the proband [6]. We propose to identify surrogate parents based on both this core and adjacent 'tails' (Figure 2), as further described below. These surrogates are termed Erdös 1 surrogates, meaning that they are one degree removed from the proband on the basis of haplotype identity [6]. The Erdös 1 surrogates of the proband are partitioned into surrogates of the paternal and maternal haplotypes. The partitioning of surrogate parents into surrogates of the paternal and maternal haplotypes is done in two ways: using pedigree information if it is available; and using a k-medoids clustering algorithm if it is not. Details on these strategies are given in Appendix A.

For the proband, inference of the phase at each locus within the paternal/maternal haplotype is attempted by stepping through the paternal/maternal surrogates until a surrogate is found that is homozygous at that locus and thus can be used to declare the phase. This is termed accessing Erdös 1 information. If a homozygote is not found at Erdös 1, the algorithm proceeds to information from surrogates at the Erdös 2 layer. Erdös 2 surrogates of a proband are surrogates who do not share a haplotype with the proband but do share a haplotype with Erdös 1 surrogates of the proband. The algorithm can continue like this for as many Erdös layers as contained within the data (Figure 1). Errors created due to incorrect surrogate identification are partially resolved by pruning from the surrogate list those surrogate parents whose haplotypes (phased by an earlier round of LRP) do not agree with the genotype of the proband (because of genotyping errors or insufficient combinatorial power to eliminate or partition surrogate parents properly), and then re-phasing all individuals using the pruned list of surrogates.

### Description of the long-range phasing and long haplotype imputation algorithm

The expansions to the LRP algorithm proposed in this research are first described individually and then followed by a description of the entire LRPHLI algorithm.

### Cores and tails

A core (Figure 2) is defined as a consecutive string of SNP loci for which phasing is being attempted. Tails (Figure 2) are defined as consecutive strings of SNP loci immediately adjacent to either end of a core. Information on homozygous loci across both core and tails are used to define surrogates. Specifically, opposing homozygotes between two individuals illustrates lack of IBD and surrogacy in that region. Tails provide additional information, thus reducing the risk of false surrogate definition, especially near the ends of the core region. Without using tails, combinatorial power may be insufficient at the ends of cores and this would result in failure to eliminate individuals that are not surrogate parents due to recombination events in these regions.

### Sparse storage and indexing of surrogate information

For each core, each individual in a dataset has potentially many surrogate parents at each Erdös level and potentially many Erdös levels (Figure 1). This information can be collapsed into a single square matrix of order equal to the number of individuals in the dataset with genotype information. For each individual, only information on Erdös 1 surrogate parents is stored explicitly, with this information identifying whether a surrogate parent is of the paternal haplotype, maternal haplotype, or of both. Surrogate parents at higher Erdös layers are thus stored implicitly. Storing an indicator as to whether a surrogate parent is paternal, maternal, or both, facilitates the use of the partitioned surrogates at the Erdös layer 1 while allowing all information to be used at higher Erdös layers. It is not necessary to partition the surrogates into maternal/paternal at higher Erdös layers [7], giving greater flexibility and power.

### Sequential long-range phasing algorithm (LRP)

The algorithm attempts to phase each locus for each proband by sequentially stepping through each Erdös layer and accessing the surrogate parents at each of these layers until a target number of surrogate parents is accessed. Once this target number of surrogate parents is accessed, the phase is determined if a proportion of these greater than a small error threshold (i.e. <10%) agrees on what the phase is. If there is disagreement amongst the surrogate parents on what is the phase, a statistical significance test can be performed.

The sequential approach ensures that even though individual surrogate parents can appear at several different Erdös levels, they are only used at their lowest Erdös level because the algorithm steps through the Erdös levels from the lowest to the highest. A restriction is included to ensure that the route to a homozygous surrogate parent can only pass through heterozygous surrogate parents. Phasing using consensus information from many surrogates reduces the impact of any individual surrogate. If information is used only from the first found homozygote, surrogate phasing error can result when false surrogates have been identified or when a surrogate contains a genotype error. Using information from multiple surrogates alleviates the need to carry out the surrogate pruning step of the Kong et al. [6] algorithm.

### Error thresholds

Identification of surrogates of a proband as individuals who show no opposing homozygotes with that proband across long strings of consecutive loci is easily disrupted by both genotype and mapping errors. This can be overcome by allowing a small percentage of opposing homozygotes without rejecting surrogacy. However this creates a new problem of increased numbers of individuals being identified as surrogates that are not in fact true surrogates. Several steps are taken to deal with this problem, including haplotype library imputation (described below), using information from multiple surrogates, and removal of surrogates that break certain rules (described below).

### Haplotype library imputation (HLI)

LRP may not work in some individuals for which there is insufficient surrogate information (e.g. due to a recombination in a gamete), or for which surrogate information is inconsistent. Some of these problems can be overcome at the end of the LRP by building a library of all unique haplotypes that LRP has found in the dataset and sequentially imputing phase for unphased individuals from this library. At each round, individuals that have one of their pair of gametes unphased can have it phased as the complement of its phased gamete via the genotype. At each round, new haplotypes that have been created through recombination can be detected and added to the library. A number of steps (described in Appendix A) are invoked during HLI to determine if a suitable haplotype exists in the library and for it to be declared as phase for the unphased gamete of the proband.

### The entire LRPHLI algorithm

Step 1: Define start point and end point of the cores and tails.

Step 2: Loop across the cores and complete the following steps for each core.

Step 2a: Identify Erdös 1 surrogate parents for each genotyped individual by looping across all SNP in the core and tails and counting the numbers of homozygote genotypes in agreement and in disagreement between the individual and all other genotyped individuals. If the count of the loci in disagreement is less than a determined threshold (e.g. 2%), an individual is taken to be a surrogate parent.

Step 2b: Partition Erdös 1 surrogates into surrogates of the paternal or maternal gamete, using the mutually exclusive strategies that are listed in Appendix A pertaining to step 2b.

Step 2c: Loop across the genotyped individuals and phase their loci based on information from the various Erdös levels as required, following strategies that are listed in the part of Appendix A that pertains to step 2c.

Step 2d: Build a haplotype library containing all completely phased haplotypes found in the dataset.

Step 2e: Impute the phase for gametes that are not completely phased by LRP by matching their phased loci to haplotypes in the haplotype library, following strategies listed in the part of Appendix A pertaining to step 2e.

Step 2f: In each proband, all phased loci are paired to create a genotype and this genotype is checked for compatibility with the genotype and if across a whole core more than 10% disagreement is found, the heterozygous loci have their phase call removed for this proband. In this case, homozygous loci are assumed to have no genotyping error and are thus phased de facto for this core of this proband.

Steps 2e and 2f are iterated until no new haplotypes are added to the library.

### Testing of performance

Performance of the LRPHLI algorithm was tested using an extensive range of simulated and real datasets.

### Simulations

In each simulation, a sample of haplotypes representing a single chromosome of 1 Morgan (100,000,000 base pairs) with the per-site mutation rate set to 10^-8 ^was created using MaCS [8], which invokes a neutral coalescent model. Two different population scenarios (Ne_100_, and Ne_1000_), which were based on the results of [9,10], were followed. Scenario Ne_100 _followed the effective population size (Ne) of Holstein cattle. Briefly, its current Ne was set to 100, the Ne 1,000 years ago to 1,200, the Ne 10,000 years ago to 4,500, and the Ne 800,000 years ago to 80,000, with gradual decreases in Ne in the intervening periods. The population with the larger Ne_1000 _could reflect a sheep breed. Its historical Ne matched that of Ne_100 _from 800,000 years ago until 2000 years ago, while from 2000 years ago until the present time, its Ne remained at 1000. Scenario Ne_100 _created approximately 56,000 segregating sites along the 1 Morgan (100,000,000 base pairs) region, while Ne_1000 _created approximately 115,000 segregating sites, with little sampling variation about these numbers. Either of these effective population sizes could possibly represent isolated human populations.

The simulated haplotypes were then dropped through eight pedigrees, which reflect a spectrum of datasets for which LRPHLI may be suitable. These included pedigrees of completely unrelated individuals, pedigrees of small, intermediate and large half-sib family structures, general livestock pedigrees, and a general pedigree of an isolated human population. Details are in Appendix B.

SNP arrays, for a single chromosome, with densities equivalent to 60,000 SNP per genome were created by randomly selecting 2,000 of the segregating sites which had minor allele frequencies of > 5% after the haplotypes had been dropped through the pedigree (60 k set). Briefly, the aims of the different simulation strategies were as follows. Pedigrees 1 to 4 tested the effect of family size, with four sire family sizes of 1, 2, 10, and 100. Pedigrees 5 to 8 were real pedigrees for livestock and human populations, which feature inbreeding loops and overlapping generations. Including and excluding genotypes on parental individuals in pedigrees 1 to 4 attempted to verify the importance of parental genotypes. Genotyping the sires rather than the last 2000 individuals in pedigrees 6 and 7 was designed to test the effect of more (sires) and less (last 2000) sparse relationships amongst genotyped individuals. To explore the effect of SNP density, a SNP array with a density of 300,000 SNP per genome (10,000 SNP per chromosome) was also created for the pedigree 1 Ne_1000 _dataset (300 k set). To explore the effect of dataset size, four subsets of pedigree 1 were created, comprising 100, 500, 1000, and all 2000 individuals. To assess the sampling error, eight replicates of pedigree 1 Ne_1000 _were carried out.

### Real datasets

Six real datasets (sheep, pig, beef cattle, dairy cattle, and human) were used to test the algorithm, all of these using SNP that had passed suitable sets of quality control criteria. The numbers of individuals genotyped, the numbers of SNP used, and the chromosome number are given in Table 1. The sheep and beef datasets had half-sib designs. The pig dataset comprised a full-sib family structure with highly related individuals and some half-sibs. The dairy dataset comprised bulls with progeny, young sires, and some bull dams. The pig dataset pedigree and data structure were actually that of pedigree 5 in the simulated datasets. The human dataset pedigree and data structure were actually that of pedigree 8 in the simulated datasets.

**Table 1:** Phasing performance for real data sets

Dataset	^1^Nb individuals	^2^Nb SNP	^3^Core/CplusT length	^4^M/E%	^5^Time	^6^% phased
Sheep chr. 4	1019	2278	100/300	1.00	3 min 39 s	98.17
Sheep chr. 5	1016	1927	100/400	1.00	5 min 1 s	97.62
Pig chr. 1	2723	3999	100/500	0.00	364 min	96.87
Beef chr. 24	2171	874	100/300	0.00	17 min 8 s	98.42
Dairy chr. 1	5057	2296	100/400	0.00	456 min	97.99
Human chr. 1	879	4472	100/300	1.00	3 min 29 s	93.73

^1^Numbers of individuals in the dataset; ^2^Numbers of SNP to be phased; ^3^Optimal core and CplusT length parameter; ^4^Optimal missing genotype/genotype error % (M/E%) error threshold parameter; ^5^Computation time measured on a 64 bit desktop with an Intel i7 3.07 GHz quad core processor running Linux was used to measure computation time; computation time includes time required to parse and summarise the data and write out the results; ^6^Percentage of alleles phased

### Phasing settings for simulated data

The simulated datasets were phased using a wide range of core and core plus tail lengths (CplusT) and using and ignoring pedigree information. CplusT length encompasses the core length plus the two tail lengths of equal size adjacent to each end of the core. For example, when the core length is 100 and the CplusT length is 100, the tail length is zero, and when the core length is 100 and the CplusT length is 200, the tail length is 50 SNP. For the 60 k SNP datasets, we varied core lengths from 100 to 2000 SNP and CplusT lengths from 100 to 2000 SNP. For the 300 k SNP dataset core lengths varied from 400 to 10000 and CplusT length from 400 to 10000 SNP.

### Phasing settings for real data

For the real datasets, core and CplusT lengths were similar to those for the 60 k simulated datasets. For each of the datasets, genotype error/missing genotype thresholds (**M/E%**) of 0%, 1%, 2%, 3%, 4%, and 5% were used and the pedigree information that was available was used.

## Results

### Phasing performance

The method performed well in terms of percentage of alleles correctly phased (**% correct**), percentage of alleles incorrectly phased (**% incorrect**), and percentage of alleles not phased (**% not phased**), across the wide spectrum of simulated scenarios when near optimal core and CplusT lengths were used. This was also the case in terms of the percentage of alleles phased (**% phased**) and % not phased across the wide spectrum of real datasets when near optimal core and CplusT lengths and M/E% thresholds were used.

The results were not subject to large sampling variation, e.g., the mean % correct for the eight independent replicates of pedigree 1 Ne_1000 _was 99.15%, with minimum and maximum values of 99.11% and 99.19%, respectively. Therefore, the summary results presented in Table 2, for the most optimal core and CplusT lengths for each simulated scenario, are based on a single replicate of each scenario. The % correct was greater than 97% and the % incorrect was less than 0.66% for all simulated scenarios, with the exception of pedigree 8, which was the smallest dataset with 879 genotyped individuals. The highest % correct (99.76) was observed for pedigree 4 with parents genotyped and Ne_1000_. The lowest % correct (95.01) was observed for pedigree 8 Ne_100_. When the % correct was high, the % incorrect was low, while when it was low, the % incorrect was still low. This shows the algorithm's robustness to error, even when the overall phasing yield (in terms of % of alleles phased) is low. The % phased was greater than 97.5% for all the real datasets (Table 1), with the exception of the human pedigree.

**Table 2:** Percentage of alleles correctly/incorrectly phased by the most optimal setings^1 ^for the simulated data sets

	Ne 100		Ne 1000	
	with pedigree	without pedigree	with pedigree	without pedigree
Pedigree 1		99.11/0.30		99.11/0.30
Pedigree 2 NPG^2^	97.85/0.43	98.49/0.63	97.73/0.29	97.88/0.39
Pedigree 2 PG^3^	98.85/0.49	99.03/0.42	99.70/0.17	99.48/0.17
Pedigree 3 NPG^2^	98.35/0.38	98.61/0.63	99.23/0.14	99.14/0.27
Pedigree 3 PG^3^	99.23/0.37	99.05/0.41	99.76/0.16	99.58/0.13
Pedigree 4 NPG^2^	98.20/0.41	98.61/0.63	98.19/0.41	98.61/0.63
Pedigree 4 PG^3^	99.35/0.31	99.29/0.32	99.74/0.20	99.59/0.15
Pedigree 5	97.59/0.42	98.28/0.60	99.30/0.30	99.31/0.22
Pedigree 6 sires	97.05/0.45	98.40/0.62	99.05/0.17	99.25/0.20
Pedigree 6 last 2000	98.24/0.39	98.24/0.39	99.34/0.20	99.42/0.26
Pedigree 7 sires	97.56/0.40	98.71/0.50	98.98/0.20	99.15/0.29
Pedigree 7 last 2000	96.86/0.46	98.40/0.66	98.85/0.20	99.34/0.26
Pedigree 8	95.01/1.10	96.67/1.39	96.02/0.57	96.36/1.01

^1^Optimal settings refer to the optimal core and CplusT lengths; core length was 100 SNP, CplusT length varied between 300 and 500 SNP; ^2^NPG indicates that this dataset did not have parents genotyped; ^3^PG indicates that this dataset had parents genotyped

Ignoring pedigree information gave better % correct than using it in ten out of the twelve comparisons possible for Ne_100_, although the differences were small (Table 2). For Ne_1000_, ignoring pedigree information gave better % correct results in seven of the eleven comparisons and gave worse results in four scenarios (each of which had a half-sib design). However pedigree information generally reduced the % incorrect (Table 2), meaning that pedigree information could be interpreted as being of marginally positive value. However differences were small in comparison to the absence of pedigree.

The algorithm was invariant to family structure. Pedigree 1 comprised unrelated individuals, while pedigrees 2, 3, and 4 comprised half-sib designs with sire family sizes of 2, 10, and 100 respectively. These pedigrees showed very small differences in the % correct (< 0.5% for Ne_100 _and < 0.65% for Ne_1000_) (Table 2) when all individuals were genotyped. Results from real livestock pedigrees confirmed these results, with very small differences in accuracy between sheep, pig and dairy cattle pedigrees.

Genotype information on parents in pedigrees 2, 3, and 4 gave slightly better results than not having it (Table 2), with differences in % correct less than 1% in all cases except for pedigree 2 Ne_1000_, for which the difference in % correct was close to 2%. It is possible that these differences are due to the reduction in size of the datasets rather than a real effect of having parents genotyped (discussed in the next section). Two genotyping strategies were applied to pedigrees 6 and 7, one which genotyped the sires in these pedigrees and another which genotyped the 2000 most recently born individuals. The difference in these strategies was very small and no consistent trend was discernable.

Pedigree 8 had the lowest number of individuals genotyped (879 individuals) and gave results that were noticeably worse than those of other datasets. To further explore the effect of dataset size, four subsets of the pedigree 1 dataset, with a random 100, 500, and 1000 individuals, and all 2000 individuals, were used. The % correct increased with increasing dataset size; the 100, 500, 1000, and 2000 individuals datasets gave % correct of 71.61%, 96.19%, 98.14%, and 99.11%, respectively, suggesting that datasets of less than 1000 individuals are too small for providing accurate phasing results.

The effect of SNP density was tested by comparing a 300 k density with the 60 k density for Pedigree 1 Ne_1000_. The results for the 300 k density were better than those of the 60 k density in terms of both % correct and % incorrect (99.11% correct and 0.30% incorrect for 60 k and 99.61% correct and 0.21% incorrect for 300 k). Similar to the 60 k density, small-sized cores and intermediate-sized CplusT gave the best results for the 300 k density, with the best results being obtained for a core of 400 SNP and a CplusT of 1200 SNP. Similar to the 60 k density, the 300 k density consistently gave very low % incorrect (< 0.62%) with all core or CplusT lengths, with the exception of those that were very short (i.e. ≤ 800 SNP). Given appropriate adjustment of core and CplusT length, the algorithm was invariant to the two SNP densities tested, with core lengths of about 5 cM and CplusT lengths of about 15 cM being close to optimal for both.

**Figure 3:**
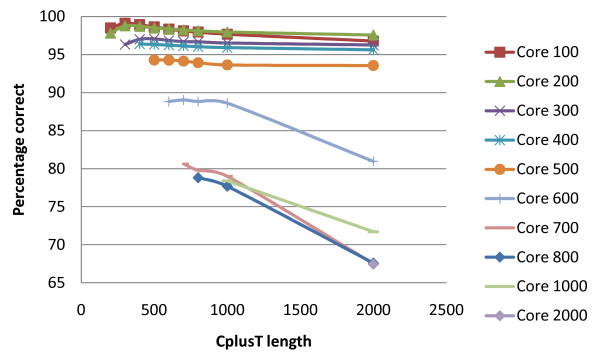
**Effect of core and CplusT lengths on the percentage of alleles correctly phased for pedigree 1 Ne_1000_**.

**Figure 4:**
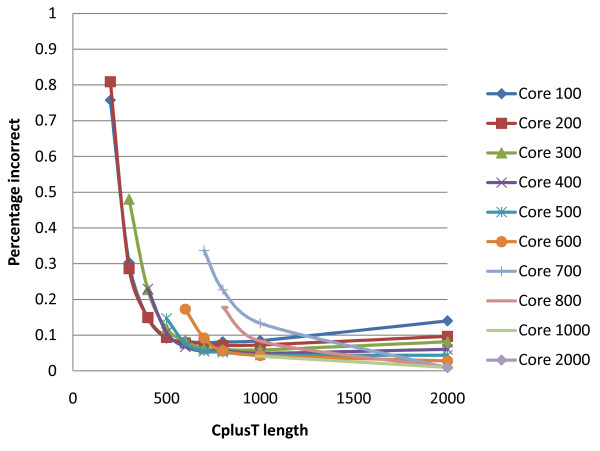
**Effect of core and CplusT lengths on the percentage of alleles correctly phased for pedigree 1 Ne_1000_**.

A wide range of core and CplusT lengths was explored for each pedigree and data scenario and for each effective population size. The observed trend across these scenarios was similar, with results for pedigree 1 Ne_1000 _being illustrative (Figures 3, 4, and 6). Very long cores and CplusT's gave lower performance in terms of % correct because very long (e.g. 100 cM) haplotypes are more varied and have a lower frequency, making both the identification of surrogates and the HLI more difficult. Extremely short CplusTs and cores suffer from a lack of combinatorial power, resulting in difficulty in partitioning and eliminating surrogates and in difficulty for the haplotype imputation step to find unique pairs of haplotypes that explain the genotype (due to there being too many compatible haplotypes). The best results, in terms of % correct, were generally obtained by using a short core (100 SNP) and an intermediate CplusT length (300 to 500 SNP). In terms of % incorrect, shorter CplusT lengths (≤200 SNP) gave by far the worst results due the lack of combinatorial power. Intermediate and longer cores gave the best results, with generally less than 0.2% error (Figure 4). Importantly, the algorithm was robust to most deviations from the optimal core and CplusT lengths within reasonably wide boundaries (Figure 6). The sharp optimal frontier in Figure 6 suggests that achieving 100% correctly phased alleles is not possible at the SNP densities tested due to lack of combinatorial power. However in Figure 6, the points tend to the bottom and to the right, effectively forming a pareto-front that constitutes a set of possible outcomes dependent on the relative emphasis on % correct and % incorrect. The sharp curve at the bottom right is fortuitous, as it shows good performance for both criteria.

**Figure 5:**
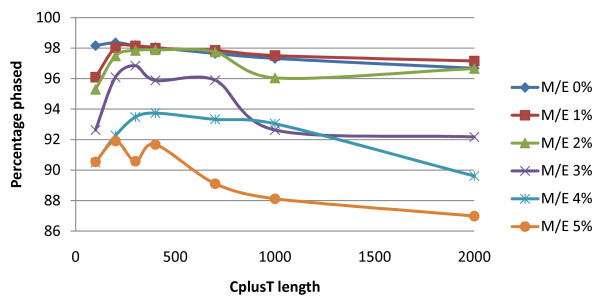
**Percentage of alleles phased for the sheep chromosome 4 dataset**. Six M/E% thresholds (0%, 1%, 2%, 3%, 4%, and 5%), a core length of 100 SNP and seven CplusT lengths were used.

**Figure 6:**
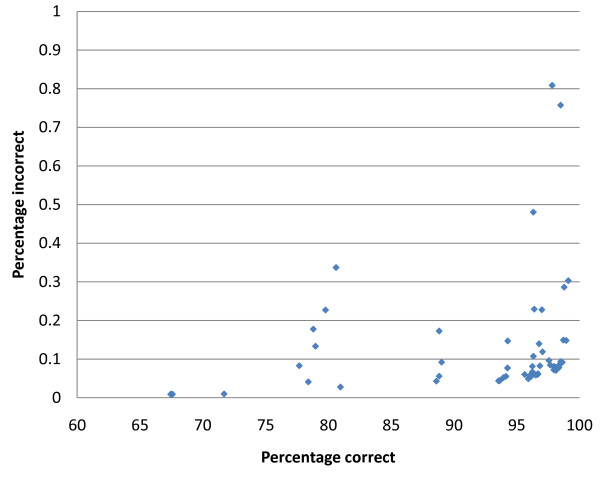
**X-Y plot for percentage incorrectly phased and percentage correctly phased for all core and CplusT lengths tested on pedigree 1 Ne_1000_**.

### Effect of the haplotype library imputation step

The benefit of the HLI step was evaluated by phasing the Ne_100 _60 k datasets for the eight pedigrees of a single replicate of simulated data using the full LRPHLI algorithm and the full algorithm with the HLI step turned off. Across the eight pedigrees, the full algorithm had an average of 97.51% correct and 0.69% incorrect, while the full algorithm with the LHI step turned off had an average of 83.15% correct and 0.97% incorrect. We do not consider that this reflects the relative performance of the Kong et al. [6] LRP algorithm compared to the LRPHLI algorithm described here because several additional steps in the LRP component of LRPHLI that were included to reduce % incorrect have a heavy penalty on the overall yield. However, LRPHLI can afford to do this because the haplotype library imputation step resurrects this lost yield.

### Computation costs

The computation time required for the different datasets for Ne_1000 _using cores of 100 SNP and CplusTs of 300 SNP ranged from less than three minutes to 321 minutes (Table 3). The trends in Table 3 and other results not shown indicate that, while increases in dataset size, relatedness/effective population size, and SNP density each increase computation time, consistent trends across all datasets and scenarios do not exist. The principle computation bottleneck was the partitioning of surrogate parents into their paternal and maternal surrogate parent clusters and this was directly related to the numbers of surrogate parents. The numbers of surrogate parents in a dataset is determined by its size, its effective population size, level of relatedness amongst its individuals, the proportion of genotype errors allowed for, and the length of the CplusTs. More related individuals are more likely to carry the same haplotypes. Shorter CplusTs are more likely to result in more surrogates because shorter haplotypes are more common than longer ones and shorter CplusTs have less combinatorial power to eliminate individuals as surrogates. These interacting factors make predictions about computation time difficult. However rules of thumb can be suggested based on the following logic. Increasing size, relatedness, and inbreeding levels increase the number of surrogate parents in a dataset, which increases the computational requirements, in some instances drastically. Increasing CplusT length reduces the number of surrogates, thus reducing the computational requirements. Higher levels of relatedness and inbreeding can sustain longer CplusT. Therefore knowledge of such features of a dataset can be used to determine suitable core and CplusT lengths to obtain a high % of phased alleles with reasonable computational costs.

**Table 3:** Computation time^1 ^required to phase 2000 ^2^SNP for the simulated Ne_1000 _data when using or ignoring pedigree information

	Number of individuals	With pedigree	Without pedigree
Pedigree 1	2000		30 min 34 s
Pedigree 2 NPG^3^	1000	4 min 3 s	3 min 3 s
Pedigree 2 PG^4^	2000	39 min 21 s	26 min 58 s
Pedigree 3 NPG^3^	1000	7 min 56 s	5 min 20 s
Pedigree 3 PG^4^	1600	18 min 23 s	17 min 11 s
Pedigree 4 NPG^3^	1000	9 min30 s	50 min 27 s
Pedigree 4 PG^4^	1510	17 min 4 s	10 min 48 s
Pedigree 5	3000	106 min 37 s	179 min 52 s
Pedigree 6 sires	2578	78 min 22 s	39 min 43 s
Pedigree 6 last 2000	2000	48 min 24 s	321 min 27 s
Pedigree 7 sires	1777	23 min 47 s	22 min 18 s
Pedigree 7 last 2000	2000	54 min 1 s	74 min 15 s
Pedigree 8	879	4 min 14 s	2 min 41 s

^1^A 64 bit desktop with an Intel i7 3.07 GHz quad core processor running Linux was used to measure computation time; computation time measured in minutes and seconds includes time required to parse and summarise the data and write out the results; ^2^using a core length of 100 SNP and a CplusT length of 300 SNP for each dataset; ^3^NPG indicates that this dataset did not have parents genotyped; ^4^PG indicates that this dataset had parents genotyped

For example, for pedigree 5 Ne_1000 _and ignoring pedigree information, the computation time was 179 minutes for a core length of 100 SNP and a CplusT length of 300 SNP and only 39 minutes for a core length of 100 SNP and a CplusT length of 500 SNP, with little difference in phasing yield. Optimal core and CplusT lengths in terms of phasing accuracy coincided with intermediate computational time requirements. Given that phasing accuracy is robust to minor changes in CplusT lengths around the optimum, the opportunity exists for using slightly longer CplusT lengths with minimal loss in phasing performance but major advantages in terms of computation time (Figure 2, 3, and 4).

Computation time is also affected by the number of SNP to be phased. For example, the pedigree 1 Ne_1000 _dataset (core 800, CplusT 1200) took 83 minutes with 300 k SNP but only 30 minutes with 60 k SNP. The number of SNP does not increase computation time linearly because greater SNP density gives greater power to eliminate surrogates, which can reduce computation time.

Using pedigree information reduced the computation time in the larger datasets, which had more relatedness amongst the genotyped individuals, because both relatedness and large data set size cause more surrogate parents (Table 3). Memory requirements were minimal; less than 0.4 gigabytes, even for the largest datasets (pedigree 5).

### Comparison to fastPHASE

The first 1000 SNP of each of the eight replicates of pedigree 1 Ne_1000 _were also phased using the default settings of fastPHASE [4]. The mean % correct across the 10 core lengths of 100 SNP across each of the eight replicates was 91.0%, with the remainder being phased incorrectly. The computation time was 13 hours for each of these datasets. In comparison, LRPHLI completed the task for twice as many SNP in these datasets in 30 minutes, 99.1% phased correctly. Although better results may be possible with fastPHASE [4] if the default settings had not been used, a very large difference would likely remain between these methods in terms of speed and accuracy.

### Real data

In the real datasets, the LRPHLI algorithm performed well in terms of % phased, % not phased, and computation time. With the exception of the human dataset, the % phased was greater than 97.5% (Table 1). The human dataset, which only had a 93.7% phased, was the smallest dataset in terms of numbers of individuals genotyped (879) and probably has a larger effective population size than the livestock datasets. The highest % phased was for the dairy cattle dataset (98.4%), which had the largest number of individuals genotyped (Table 1). Across the large spectra of core and CplusT lengths that were tested, the highest % phased in each of the datasets was obtained with relatively short core lengths and intermediate CplusT lengths (results not shown because the patterns were very similar to those for the simulated datasets).

The highest % phased was obtained with low M/E% thresholds (e.g. 0 or 1%). The lower % phased obtained with higher thresholds was probably due to ambiguity in eliminating or partitioning surrogates and a consequential conflict in what the surrogates suggest the phase is, which results in an uncalled phase for such an allele. For shorter CplusT lengths, the % phased tended to be lower with a 1% M/E% threshold than with a 0% M/E% threshold but there was little difference between these thresholds for intermediate CplusT lengths, because intermediate CplusTs lengths were better able to compensate for ambiguity introduced by accounting for M/E% threshold than the short CplusTs lengths. This suggests that in datasets with missing genotypes or genotyping errors, slightly longer CplusTs should be used in conjunction with a low M/E% threshold.

## Discussion

This study investigated an extended version of long-range phasing, and combined it with haplotype library imputation, resulting in an accurate and fast phasing method. Phasing results of up to 99.5% correct in simulated data and up to 98.5% phased in real data were achieved. Computation time was very tractable even for datasets of 5000 genotyped individuals and 300 k SNP densities. This new LRPHLI algorithm outperformed fastPHASE manyfold in both accuracy and speed, making the routine phasing of datasets for genome-wide association studies and genomic selection feasible. For much larger datasets (e.g. 400,000 individuals), a similar framework to the one presented here could be used but with some additional steps [11]. This involves first phasing small (random or strategic) subsets of the large dataset using the complete LRPHLI algorithm described herein, followed by phasing the remaining individuals using the haplotype library imputation step alone. This framework avoids much of the task of partitioning surrogates parents into their paternal and maternal clusters, which is the primary computation bottleneck in LRPHLI.

The LRPHLI algorithm adds a number of components to the original Kong et al. [6] LRP algorithm. These include pedigree and pedigree free strategies for partitioning of surrogate parents, the concepts of cores and tails for more accurate surrogate parent definition, steps to avoid errors (e.g. removal of surrogate parents who are not different enough, using information from multiple surrogates), steps to avoid low yields where recombination events create new haplotypes (haplotype library imputation), and measures to handle missing genotype/genotype error. It is the combination of these additions that give the algorithm its robust performance.

A drawback of our proposed algorithm is that chromosomes are partitioned into cores that are generally not aligned with respect to each other. Strategies to align cores along a chromosome include the use of pedigree information (either explicitly or as part of a segregation analysis), using overlapping cores to step across a chromosome, or aligning based on the similarity between clusters of surrogates for adjacent cores. This latter strategy could also be used to speed up the partitioning of surrogate steps (i.e. good starting values), while the segregation analysis strategy could also be used to impute dense genotype or sequence information in sparsely genotyped pedigrees [11], where scarcity both refers to low density genotyping arrays and to completely ungenotyped individuals in the pedigree. Imputation of genotype data is currently part of many national genetic evaluation systems, e.g. [12-15]. A major advantage of the LRPHLI algorithm for phasing and of the LRPHLI algorithm when combined with segregation analysis for imputation of genotypes, is that other phasing and imputation methods [13-16] require more restrictive genotyping strategies, e.g. that both parents are densely genotyped. The LRPHLI algorithm can transfer information upwards, downwards and sideways in a pedigree, similar to the algorithm outlined by VanRaden [12].

The haplotypes obtained from the LRPHLI algorithm can be used directly in association studies. Because the small error percentages (e.g. 0.5% incorrect) create pairs or clusters of haplotypes that are very similar (e.g. 99.5% identical) but that are identified as different by LRPHLI, clustering these haplotypes into groups of similar haplotypes could be useful.

## Conclusions

The long-range phasing and haplotype library imputation algorithm performs well in both simulated (up to 99.5% correct) and real datasets (up to 98.5% phased) and had low computation requirements, making the routine phasing of high density SNP chip datasets feasible. The phasing performance was robust to effective population size, pedigree and data structure, and SNP density. The best results were obtained with short core lengths (e.g. 100 SNP for 60 k SNP density and 400 SNP for 300 k SNP density) and intermediate CplusT lengths (e.g. 300 SNP for 60 k SNP density and 1200 SNP for 300 k SNP density). The partitioning of surrogate parents into their paternal and maternal clusters was the principle computation bottleneck and was affected by the dataset size, effective population size, and the degree of relatedness among the individuals. Ignoring pedigree information had a negligible effect on the phasing performance but did increase computation time in the larger datasets.

## AlphaPhase

The algorithm is implemented in a new software package called AlphaPhase, which was written in Fortran 95. AlphaPhase is controlled by a parameter file and is freely available for research purposes from http://sites.google.com/site/hickeyjohn/alphaphase.

## Appendix A

### Detailed description of key steps in the LRPHLI algorithm

Step 2b: Partition Erdös 1 surrogates into surrogates of the paternal or maternal gamete using the following mutually exclusive strategies, which are listed in order of the precedence:

i. If both parents of a proband are genotyped, paternal (maternal) surrogate parents are identified as those surrogate parents who are surrogate parents of the father (mother) but not of the mother (father). The remaining surrogates are banned from use at any Erdös level for this proband.

ii. If the father of a proband is genotyped, surrogate parents that are also surrogate parents of the father are placed in the paternal surrogate parent cluster. Maternal surrogate parents are identified by first choosing a dummy mother. A dummy mother is chosen by looping through the remaining surrogate parents until the surrogate parent with greatest numbers of opposing homozygote loci with the father is found and if this individual has a count of opposing homozygotes greater than 10% of the SNPs in the core and tail in disagreement with the father it is taken to be the dummy mother. The dummy mother is assumed to be a maternal surrogate parent. The remaining surrogate parents are iteratively added to the maternal surrogate parent cluster if they are not surrogate parents of one of the paternal surrogate parents and are surrogate parents of the dummy mother or one of the subsequently identified maternal surrogate parents.

iii. If the mother of a proband is genotyped the partitioning is similar to that described in ii, but for the mother.

iv. If neither parent is genotyped but pedigree information is available, a dummy father (mother) is identified as a surrogate parent who has a coefficient of relationship of greater than a set threshold with the sire (dam) of the proband and of less than a certain threshold with the dam (sire) of the proband. Paternal (maternal) surrogate parents are identified as those who are surrogate parents of the proband and the dummy father (mother) but not the dummy mother (father).

v. If the proband has offspring which are genotyped, one of the offspring is chosen to be a dummy father (with arbitrary gender allocation for simple presentation), the dummy father and other offspring which are also surrogates of the dummy father are arbitrarily labelled the paternal surrogate parents. A dummy mother is chosen by looping through the remaining surrogate parents until a surrogate parent who is not a surrogate parent of the dummy father and has a count of opposing homozygotes greater than 10% of the SNPs in the core and tail in disagreement with the dummy father is found. The dummy mother is assumed to be a maternal surrogate parent. The remaining surrogate parents are iteratively added to the paternal (maternal) surrogate parent cluster if they are not surrogate parents of one of the maternal (paternal) surrogate parents and are surrogate parents of the dummy father (mother) or one of the subsequently identified paternal (maternal) surrogate parents.

vi. If there is no pedigree information available or the surrogate parents have not been clustered by any of the preceding methods, the k-medoids clustering algorithm is invoked to divide the surrogate parents into two clusters which are arbitrarily labelled paternal and maternal. The clustering algorithm works with a symmetrical matrix of dimension equal to the number of surrogate parents and elements indicating surrogacy amongst the surrogate parents, with 1's denoting surrogacy and 0's otherwise. The two clusters are initialised by ascribing the first surrogate parent found and all surrogate parents who are also surrogate parents of this surrogate parent to one cluster and all surrogate parents who are not surrogate parents of this surrogate parent to the other cluster. The medoids of the two clusters are represented by two vectors of length number of surrogate parents, with each element containing the sum of 1's for that location divided by the square of the number of individuals currently in the cluster. The distance between each surrogate parent and the medoid of both clusters is calculated as the average absolute deviation of the elements in the row of the symmetrical matrix from the corresponding element in the medoid vector. Surrogate parents are moved to the alternative cluster if the distance to the alternative clusters medoid is less than the distance to its current medoid. The medoids are recalculated when a surrogate parent moves clusters and the algorithm proceeds until convergence or the maximum permitted iterations is reached. The maximum number of iterations permitted is set to the number of surrogate parents of the proband.

Step 2c: Loop across all individuals carrying out the following steps:

i. Individuals, which are not Erdös 1 surrogates of the proband but whose count of opposing homozygote loci with the proband only exceeds the threshold for inclusion as a surrogate parent by a count of less than the average difference of those genotyped individuals that are not identified as surrogate parents, are banned from use at any Erdös level for this proband.

ii. Long-range phases all loci within the core for both the paternal and maternal gametes by sequentially stepping from Erdös level 1 to the maximum Erdös level, until one of the stopping criteria is met. As surrogates are accessed, they are banned from use at higher Erdös levels. nTarget is the targeted number of homozygote surrogate parents (including the proband itself if it is homozygous) across which information about phase is accumulated. Once nTarget is reached the stepping through the Erdös levels stops, phase is declared if a proportion greater than an error threshold (e.g. 10% error) of the nTarget surrogate parents indicate that phase is in one direction, or a statistically significant proportion of the surrogates indicates phase is on one direction rather than the other. If the search through all surrogate parents at all Erdös levels does not yield nTarget homozygous surrogate parents phase is declared if all surrogate parents which are found to be homozygous at this locus indicate that phase is in one direction, or a statistically significant proportion of the surrogate parents which are found indicate phase is on one direction rather than the other.

iii. Check across all loci in the core that the phased gametes are compatible with the genotype. If the count of incompatible loci exceeds a threshold (e.g. 5%), all loci which are heterozygous or missing are declared unphased at this stage.

Step 2e: Impute phase for gametes which are not completely phased by LRP by matching their phased loci to haplotypes in the haplotype library. This matching is carried out by one of the following steps which are listed in the order of precedence:

i. If one of a proband's gametes is completely phased the library is searched for candidate haplotypes which, when paired with the proband's completely phased gamete can explain the proband's genotype given a certain tolerance for error along the core. If one candidate haplotype is identified it is assumed to explain the phase of the unphased loci in the core. If more than one candidate haplotype is identified, phase for the unphased loci is determined at the loci where the candidate haplotypes agree. If no candidate haplotype is identified phase for the unphased loci is assumed to be the complement of the phased loci of the phased gamete via the genotype and this new haplotype is added to the library.

ii. If neither of the proband's gametes is completely phased the library is searched for candidate haplotypes which agree given a certain tolerance for error with the phased loci along one of the proband's gametes. If one candidate haplotype is found for the paternal gamete and one candidate haplotype is found for the maternal gamete these are paired and if the sum agrees with the genotype at all loci given a certain tolerance for error this pair of haplotypes is assumed to explain the phase of the unphased loci in the core. If one candidate haplotype is found for the paternal gamete and more than one candidate haplotype is found for the maternal gamete the paternal candidate and each of the maternal haplotypes are paired and if only a single of these pairs agrees with the genotype at all loci given a certain tolerance for error this pair of haplotypes is assumed to explain the phase of the unphased loci in the core. If more than one of these pairs are compatible, phase for the unphased loci is determined at the loci where the candidate haplotypes agree. A similar step is carried out where one candidate haplotype is found for the maternal gamete and more than one candidate haplotype is found for the paternal gamete.

iii. If more than one candidate haplotype is found for the paternal gamete and no candidate haplotype is found for the maternal gamete, phase for the unphased loci of the paternal gamete is determined at the loci where the candidate haplotypes agree. A similar step is carried out where more than one candidate haplotype is found for the maternal gamete and no candidate haplotypes are found for the paternal gamete.

iv. Probands which are still not completely phased but have more than one candidate haplotype identified have these candidates exhaustively paired. If only a single pair explains phase given a certain tolerance for error this pair of haplotypes is assumed to explain the phase of the unphased loci in the core. If more than 1 pair explains phase given a certain tolerance for error a k-medoids type clustering algorithm (described above) is invoked to make two clusters out of the candidate haplotypes. All loci in the core of the proband being worked on have their previously determined phase information deleted, the homozygous loci for this proband are assumed to have no genotyping error and are thus re-phased *de facto *and phase for heterozygous loci for this proband is determined at the loci where all the candidate haplotypes within a cluster agree. It is assumed that one of the clusters represents the maternal haplotype and the other cluster represents the paternal haplotype.

## Appendix B

### Detailed description of the structures and genotyping strategies for the eight pedigrees used to test performance of the algorithm

#### Pedigree structure

Pedigree 1 comprised 2,000 "unrelated" individuals with unknown parents. Pedigree 2 comprised 2,000 individuals from two generations (500 sires, 500 dams, and 1000 offspring). Pedigree 3 comprised 1,600 individuals from two generations (100 sires, 500 dams, and 1000 offspring). Pedigree 4 comprised 1,510 individuals from two generations (10 sires, 500 dams, and 1000 offspring). Pedigree 5 was a real pedigree of a commercial pig population (courtesy of the Pig Improvement Company) comprising 6,242 individuals, across 16 generational tiers with the largest sire family size being 66 individuals. Pedigree 6 was obtained from an Australian terminal sire sheep breed (courtesy of Sheep Genetics Australia) comprising 9,557 individuals across 21 generational tiers with the largest sire family size being 142 individuals. Pedigree 7 represented the genotyped Australian Holstein sires (courtesy of Australian Dairy Herd Improvement Scheme) comprising 20,792 individuals, across 13 generational tiers with the largest sire family size being 528 individuals. Pedigree 8 was a human pedigree of genotyped individuals from the ORCADES study in the Orkney Islands, Scotland [17], comprising 12,294 individuals across 12 generational tiers with the largest sire family being 8 individuals.

#### Genotyping strategy

For pedigree 1, all individuals were genotyped. For pedigrees 2, 3, and 4, either all individuals or only second generation offspring were assumed genotyped. For pedigree 5, the genotypes for 3,000 individuals were assumed known, reflecting the same animals genotyped as in the actual Pig Improvement Company dataset. For pedigrees 6 and 7, two genotyping strategies were implemented, with either the last 2,000 individuals of the pedigree or the sires (2,578 sires in pedigree 6 and 1,777 sires in pedigree 7) genotyped. For pedigree 8, we used data from all 749 individuals for which genotypes were available in the Orkney Complex Disease Study (ORCADES).

## Competing interests

The authors declare that they have no competing interests.

## Authors' contributions

JMH and BPK designed the algorithm and experiments to test it. JHJW and BT advised on the design of the experiments to test the algorithm. ND advised on the k-medoids clustering. JMH, BPK, and JHJW wrote the manuscript. JFW contributed the ORCADES data. All authors read and approved the final manuscript.

## References

[B1] Kong A Steinthorsdottir V Masson G Thorleifsson G Sulem P Besenbacher S Jonasdottir A Sigurdsson A Kristinsson KT Jonasdottir A Frigge ML Gylfason A Olason PI Gudjonsson SA Sverrisson S Stacey SN Sigurgeirsson B Benediktsdottir KR Sigurdsson H Jonsson T Benediktsson R Olafsson JH Johannsson OT Hreidarsson AB Sigurdsson G DIAGRAM Consortium Ferguson-Smith AC Gudbjartsson DF Thorsteinsdottir U Stefansson K Parental origin of sequence variants associated with complex diseases Nature 2009 462 868 874 10.1038/nature08625 20016592 PMC3746295

[B2] Howie BN Donnelly P Marchini J A flexible and accurate genotype imputation method for the next generation of genome-wide association studies Plos Genet 2009 5 e1000529 10.1371/journal.pgen.1000529 19543373 PMC2689936

[B3] Li Y Willer C Sanna S Abecasis G Genotype imputation Ann Rev Genomics Hum Genet 2009 10 387 406 10.1146/annurev.genom.9.081307.164242 PMC2925172 19715440

[B4] Scheet P Stephens M A fast and flexible statistical model for large-scale population genotype data: applications to inferring missing genotypes and haplotypic phase Am J Hum Genet 2006 78 629 644 10.1086/502802 16532393 PMC1424677

[B5] Browning SR Browning BL Rapid and accurate haplotype phasing and missing-data inference for whole-genome association studies by use of localized haplotype clustering Am J Hum Genet 2007 81 1084 1097 10.1086/521987 17924348 PMC2265661

[B6] Kong A Masson G Frigge ML Gylfason A Zusmanovich P Thorleifsson G Olason PI Ingason A Steinberg S Rafnar T Sulem P Mouy M Jonsson F Thorsteinsdottir U Gudbjartsson DF Stefansson H Stefansson K Detection of sharing by descent, long-range phasing and haplotype imputation Nat Genet 2008 40 1068 1075 10.1038/ng.216 19165921 PMC4540081

[B7] Kinghorn BP Hickey JM van der Werf JHJ A recursive algorithm for long range phasing of SNP genotypes Proceedings of the 18th conference of the Association for the Advancement of Animal Breeding and Genetics: 28 September-1 October2009; Barossa Valley 2009 76 79

[B8] Chen GK Marjoram P Wall JD Fast and flexible simulation of DNA sequence data Genome Res 2009 19 136 142 10.1101/gr.083634.108 19029539 PMC2612967

[B9] Villa-Angulo R Matukumalli LK Gill CA Choi J Van Tassell CP Grefenstette JJ High-resolution haplotype block structure in the cattle genome BMC Genetics 2009 10 19 10.1186/1471-2156-10-19 19393054 PMC2684545

[B10] MacEachern S Hayes BJ McEwan J Goddard ME An examination of positive selection and changing effective population size in Angus and Holstein cattle populations *(Bos taurus) *using a high density SNP genotyping platform and the contribution of ancient polymorphism to genomic diversity in domestic cattle BMC Genomics 2009 10 181 10.1186/1471-2164-10-181 19393053 PMC2681480

[B11] Hickey JM Kinghorn BP Cleveland M Tier B van der Werf JHJ Recursive long range phasing and long haplotype library imputation: application to building a global haplotype library for Holstein cattle Proceedings of the 9th World Congress on Genetics Applied to Livestock Production: 1-6 August 2010; Leipzig 2010 pdf 09-34

[B12] VanRaden PM Genomic evaluations with many more genotypes and phenotypes Proceedings of the 9th World Congress on Genetics Applied to Livestock Production: 1-6 August 2010; Leipzig 2010 pdf 09-27

[B13] Daetwyler HD Wiggans GR Hayes BJ Woolliams JA Goddard ME Imputation of missing genotypes from sparse to high-density using long-range phasing Proceedings of the 9th World Congress on Genetics Applied to Livestock Production: 1-6 August 2010; Leipzig 2010 pdf 05-39 10.1534/genetics.111.128082 PMC3176129 21705746

[B14] Zhang Z Druet T Marker imputation with low-density marker panels in Dutch Holstein cattle J Dairy Sci 2010 93 5487 5494 10.3168/jds.2010-3501 20965364

[B15] Weigel KA Van Tassell CP O'Connell JR VanRaden PM Wiggans GR Prediction of unobserved single nucleotide polymorphism genotypes of Jersey cattle using reference panels and population-based imputation algorithms J Dairy Sci 2010 93 2229 2238 10.3168/jds.2009-2849 20412938

[B16] Habier D Fernando RL Dekkers JCM Genomic selection using low-density marker panels Genetics 2009 182 343 353 10.1534/genetics.108.100289 19299339 PMC2674831

[B17] McQuillan R Leutenegger AL Abdel-Rahman R Franklin CS Pericic M Barac-Lauc L Smolej-Narancic N Janicijevic B Polasek O Tenesa A Macleod AK Farrington SM Rudan P Hayward C Vitart V Rudan I Wild SH Dunlop MG Wright AF Campbell H Wilson JF Runs of homozygosity in European populations Amer J Hum Genet 2008 83 359 372 10.1016/j.ajhg.2008.08.007 18760389 PMC2556426

